# Supplementary material for: Nursing students’ self-efficacy in lifestyle counselling: Associations with learning methods
Source: PLoS One. 2025 Sep 10;20(9):e0330369. doi: 10.1371/journal.pone.0330369 (PMC12422515; doi:10.1371/journal.pone.0330369)
Supplement: S1 File — (PDF) [file pone.0330369.s001.pdf]

Self-Efficacy in Lifestyle Counselling scale – SELC 20+20

The lifestyle habits (tobacco, alcohol, physical activity and eating habits) are of great importance both for health promotion, as well as prevention and treatment of non-communicable diseases. The questionnaire is about self-efficacy in your knowledge and ability to counsel persons about their lifestyle.

For each question, choose how confident you are in your theoretical **KNOWLEDGE** and practical **ABILITY** to counsel persons within the respective lifestyle habits.	**Circle <u>one</u> number based on the following answer options:**<br><br>0. I am very insecure in my…<br>1. I am insecure in my…<br>2. I am sure of my…<br>3. I am very sure of my…								
	… confidence in theoretical **knowledge** about/to…				… confidence in practical **ability** about/to…				
**Tobacco/Nicotine**									
1. Identification of tobacco/nicotine use	0	1	2	3		0	1	2	3
2. Health effects of tobacco/nicotine use	0	1	2	3		0	1	2	3
3. Assessment of motivation for tobacco/nicotine cessation	0	1	2	3		0	1	2	3
4. Advice about tobacco/nicotine	0	1	2	3		0	1	2	3
5. Motivational strategies for tobacco/nicotine cessation	0	1	2	3		0	1	2	3
**Alcohol**									
6. Identification of alcohol consumption	0	1	2	3		0	1	2	3
7. Health effects of alcohol consumption	0	1	2	3		0	1	2	3
8. Assessment of motivation for decreased alcohol consumption	0	1	2	3		0	1	2	3
9. Advice about alcohol	0	1	2	3		0	1	2	3
10. Motivational strategies for decreased alcohol consumption	0	1	2	3		0	1	2	3
**Physical activity**									
11. Identification of physical activity	0	1	2	3		0	1	2	3
12. Health effects of physical activity	0	1	2	3		0	1	2	3
13. Assessment of motivation for physical activity	0	1	2	3		0	1	2	3
14. Advice about physical activity	0	1	2	3		0	1	2	3
15. Motivational strategies for physical activity	0	1	2	3		0	1	2	3
**Eating habits**									
16. Identification of eating habits	0	1	2	3		0	1	2	3
17. Health effects of eating habits	0	1	2	3		0	1	2	3
18. Assessment of motivation for healthier eating habits	0	1	2	3		0	1	2	3
19. Advice about eating habits	0	1	2	3		0	1	2	3
20. Motivational strategies for healthy eating habits	0	1	2	3		0	1	2	3

Thank you for your participation!

© 2024 Sara Alenius, Marie Rask, Petra Nilsson Lindström, Marie Nilsson, Lina Behm. The instrument may only be used in its entirety and with reference to the authors.
