# Supplementary material for: Nursing students’ self-efficacy in lifestyle counselling: Associations with learning methods
Source: PLoS One. 2025 Sep 10;20(9):e0330369. doi: 10.1371/journal.pone.0330369 (PMC12422515; doi:10.1371/journal.pone.0330369)
Supplement: S1 Table — Demographic questions. (PDF) [file pone.0330369.s002.pdf]

S1 Table. Bivariate associations of self-efficacy in knowledge and ability scores (*n* = 257). Demographic questions.

		**Knowledge** total score (0-60)		**Ability** total score (0-60)	
	n (%)	Mean (SD)		Mean (SD)	*p*-value
**Total**	257 (100)	37.22 (6.77)		35.91 (7.30)	**< 0.001** [a]
Independent variables	*n* (%)	Mean (SD)	*p*-value	Mean (SD)	*p*-value
**Age** (years)			0.071 [b]		**0.034** [b]
≤25	134 (52.1)	36.49 (6.07)		34.98 (6.88)	
>26	123 (47.9)	38.02 (7.39)		36.92 (7.63)	
**Sex**			0.196 [b]		**0.040** [b]
Women	229 (89.1)	37.03 (6.64)		35.58 (7.17)	
Men	28 (10.9)	38.79 (7.66)		38.57 (7.92)	
**Educational level**			0.437 [b]		0.718 [b]
Upper secondary school	225 (87.5)	37.10 (6.57)		35.84 (7.06)	
University degree	32 (12.5)	38.09 (8.09)		36.34 (8.92)	
**Previous health care education**			0.165 [b]		0.291 [b]
Yes	98 (38.1)	37.97 (7.39)		36.52 (7.97)	
No	159 (61.9)	36.76 (6.33)		35.53 (6.84)	
**Semester**			0.455 [c]		0.471 [c]
2	63 (24.5)	35.81 (8.64)		34.46 (9.36)	
3	74 (28.8)	37.73 (5.42)		36.49 (5.73)	
4	40 (15.6)	37.60 (6.63)		35.85 (7.31)	
5	59 (23)	37.75 (5.44)		36.61 (6.28)	
6	21 (8.2)	37.48 (8.22)		36.33 (7.88)	

SD Standard Deviation; [a] Paired samples t-test; [b] Independent samples t-test; [c] One-way between-groups Anova. Bolded p-values indicate statistical significance ($p < 0.05$).
