# Supplementary material for: Nursing students’ self-efficacy in lifestyle counselling: Associations with learning methods
Source: PLoS One. 2025 Sep 10;20(9):e0330369. doi: 10.1371/journal.pone.0330369 (PMC12422515; doi:10.1371/journal.pone.0330369)
Supplement: S2 Table — Learning methods. (PDF) [file pone.0330369.s003.pdf]

S2 Table. Bivariate associations of self-efficacy in knowledge and ability scores (*n* = 257). Learning methods.

	**Knowledge** total score (0-60)			**Ability** total score (0-60)		
Independent variables	*n* (%)	Mean (SD)	*p*-value	*n* (%)	Mean (SD)	*p*-value
**Own search for knowledge**			**< 0.001** [b]			**< 0.001** [b]
Not at all	2 (0.8)	35.00 (14.14)		6 (2.3)	26.00 (11.21)	
To a low degree	41 (16)	33.00 (5.47)		52 (20.2)	31.87 (5.01)	
To a high degree	135 (52.5)	36.67 (5.96)		143 (55.6)	36.05 (6.59)	
To a very high degree	79 (30.7)	40.42 (7.14)		56 (21.8)	40.36 (7.41)	
**Personal experiences**			**< 0.001** [b]			**< 0.001** [b]
Not at all	6 (2.3)	34.50 (3.21)		10 (3.9)	29.60 (6.50)	
To a low degree	47 (18.3)	33.34 (4.98)		54 (21)	31.96 (5.77)	
To a high degree	124 (48.2)	37.16 (6.66)		118 (45.9)	35.82 (6.96)	
To a very high degree	80 (31.1)	39.80 (6.93)		75 (29.2)	39.72 (6.87)	
**Theoretical knowledge through education**			**< 0.001** [b]			**< 0.001** [b]
Not at all	7 (2.7)	37.14 (6.52)		12 (4.7)	32.17 (10.15)	
To a low degree	43 (16.7)	33.81 (7.43)		46 (17.9)	31.24 (7.07)	
To a high degree	124 (48.2)	36.23 (5.69)		132 (51.4)	35.70 (6.09)	
To a very high degree	83 (32.3)	40.48 (6.68)		67 (26.1)	40.19 (6.71)	
**Observed lifestyle counselling**			**< 0.001** [b]			**< 0.001** [b]
Not at all	26 (10.1)	35.23 (6.11)		28 (10.9)	32.18 (8.14)	
To a low degree	66 (25.7)	35.21 (7.36)		78 (30.4)	33.86 (6.74)	
To a high degree	106 (41.2)	37.34 (5.25)		92 (35.8)	36.51 (6.39)	
To a very high degree	59 (23)	40.14 (7.76)		59 (23)	39.44 (7.37)	
**Experience in lifestyle counselling**			**< 0.001** [b]			**< 0.001** [b]
Not at all	28 (10.9)	33.96 (7.54)		29 (11.3)	30.97 (8.25)	
To a low degree	71 (27.6)	35.35 (5.75)		63 (24.5)	34.11 (6.77)	
To a high degree	96 (37.4)	37.60 (6.28)		97 (37.7)	36.32 (6.30)	
To a very high degree	62 (24.1)	40.24 (7.01)		68 (26.5)	39.09 (7.18)	
**Receiving feedback on counselling**			**< 0.001** [b]			**< 0.001** [b]
Not at all	33 (12.8)	33.39 (7.88)		34 (13.2)	31.06 (8.90)	
To a low degree	78 (30.4)	36.17 (5.83)		70 (27.2)	34.86 (6.39)	
To a high degree	81 (31.5)	37.40 (5.39)		87 (33.9)	35.98 (5.97)	
To a very high degree	65 (25.3)	40.22 (7.53)		66 (25.7)	39.42 (7.28)	

[b] One-way between-groups Anova. Bolded p-values indicate statistical significance ($p < 0.05$).
