# Supplementary material for: Nursing students’ self-efficacy in lifestyle counselling: Associations with learning methods
Source: PLoS One. 2025 Sep 10;20(9):e0330369. doi: 10.1371/journal.pone.0330369 (PMC12422515; doi:10.1371/journal.pone.0330369)
Supplement: Attachment — Submitted filename: Response_to_Reviewers.docx [file pone.0330369.s005.docx]

**Title:** Nursing students' self-efficacy in lifestyle counselling and its association with self-assessed learning methods.

**Manuscript ID:** PONE-D-25-19531.

**Authors:** Sara Alenius, Marie Rask, Albert Westergren, Petra Nilsson Lindström, Marie Nilsson, Lina Behm.

**Date:** 4 July 2025

Dear Editor,

We thank you and the reviewers for your time and thoughtful feedback. We have carefully revised the manuscript in accordance with the comments provided. Below, we present a detailed point-by-point response to each comment. For clarity, please see the version of the manuscript with tracked changes.

Sincerely,

Sara Alenius

On behalf of the authors

| **Journal Requirements** | **Answer** |
| --- | --- |
| 1. Please ensure that your manuscript meets PLOS ONE's style requirements, including those for file naming. | Thank you for the reminder. We have carefully revised the manuscript and associated files to ensure compliance with PLOS ONE’s style requirements, including file naming. Please let us know if any additional adjustments are needed. |
| 2. Please provide an amended statement that declares *all* the funding or sources of support (whether external or internal to your organization) received during this study, as detailed online in our guide for authors at <http://journals.plos.org/plosone/s/submit-now>.  Please also include the statement “There was no additional external funding received for this study.” in your updated Funding Statement.  Please include your amended Funding Statement within your cover letter. We will change the online submission form on your behalf. | Thank you for your comment. We have updated the Funding Statement. The revised statement, included in the cover letter, reads as follows:  **Funding Statement:**  This study was supported by Kristianstad University. The funder had no role in study design, data collection and analysis, decision to publish, or preparation of the manuscript. There was no additional external funding received for this study.  We hope this satisfies the journal’s requirements. Thank you for your guidance. |
| 3. We noted in your submission details that a portion of your manuscript may have been presented or published elsewhere. “This study used the same data as a previously published study (doi: 10.1186/s12955-024-02236-z, attached as a Related Manuscript). However, this study contains additional data about learning methods and had a completely different analysis than the previous study”.  Please clarify whether this [conference proceeding or publication] was peer-reviewed and formally published. If this work was previously peer-reviewed and published, in the cover letter please provide the reason that this work does not constitute dual publication and should be included in the current manuscript. | Thank you for raising this important point.  The related study (doi: 10.1186/s12955-024-02236-z), which we referenced in the submission as a Related Manuscript, was peer-reviewed and published in BMC Health and Quality of Life Outcomes. That publication focused specifically on the development and psychometric properties of the instrument *Self-Efficacy in Lifestyle Counselling scale (SELC 20 + 20)* using Rasch analysis. The present manuscript addresses a different set of research questions, applies a different analysis, and presents novel findings regarding learning methods and self-efficacy development among nursing students. No overlapping hypotheses, tables, figures, or interpretations of results are reused between the two papers.  Therefore, we believe this submission does not constitute a duplicate or redundant publication, as it represents a distinct and original contribution to the literature. |
| 4. We note that this data set consists of interview transcripts. Can you please confirm that all participants gave consent for interview transcript to be published? | Thank you for your comment. We would like to clarify that this study did not involve interviews. The data were collected exclusively through questionnaires, as part of a quantitative study design. Accordingly, no interview data exist, and no participant consent for publication of transcripts was needed or obtained, as such data were never collected.  We hope this clarifies the nature of the dataset. |
| 5. Please review your reference list to ensure that it is complete and correct. If you have cited papers that have been retracted, please include the rationale for doing so in the manuscript text, or remove these references and replace them with relevant current references. Any changes to the reference list should be mentioned in the rebuttal letter that accompanies your revised manuscript. If you need to cite a retracted article, indicate the article’s retracted status in the References list and also include a citation and full reference for the retraction notice. | Thank you for your comment regarding the reference list. We have carefully reviewed all references to ensure they are complete and formatted in accordance with the journal’s guidelines and the Vancouver style. During this process, we also checked for any retracted articles.   - No retracted references were identified among the cited sources. - The reference list has been updated for accuracy, including minor corrections to author names, journal abbreviations, and article identifiers (e.g., page ranges and DOIs or article numbers where applicable). - Where applicable, official English translations of institutional authors and titles have been used (e.g., for Swedish government publications). - URLs have been verified for functionality. |
| **Reviewer comment** | **Answer** |
| **Reviewer #1:** The manuscript makes a meaningful contribution and demonstrates methodological rigor and clarity. Addressing the minor revisions would improve clarity and impact, especially regarding educational practice and generalizability. | Thank you very much for your thoughtful and encouraging feedback. We are pleased that the manuscript is seen as a meaningful contribution with methodological rigor and clarity. We appreciate your suggestions for improvement and have addressed the minor revisions to enhance clarity and impact, particularly in relation to educational practice and generalizability. |
| **Title:** Consider:  *“Nursing Students’ Self-Efficacy in Lifestyle Counselling: Associations with Learning Methods”* (More concise, but still informative.) | Thank you for the helpful suggestion regarding the manuscript title. We agree that the proposed version is more concise while maintaining clarity and relevance. The title has been revised accordingly. Please see page 1, lines 1–4 of the revised manuscript. |
| **Abstract** under methods, **Suggestion:** Include a key statistic from the regression model (e.g., R² values or effect sizes). | Thank you for the helpful comment. We have revised the abstract to include the R² values from the regression analyses. The updated information can be found in the results portion of the abstract (page 3, lines 49 and 52). |
| **Abstract:** Clarify what "SELC20+20" stands for directly in the abstract. | Thank you for the comment. We agree that clarity is important for readers unfamiliar with the scale. The abstract already includes the full name of the instrument - “the self-efficacy in lifestyle counselling scale (SELC20+20)” **-** at first mention. We hope this formulation sufficiently clarifies the abbreviation. Please see the method section of the abstract (page 2, line 38). |
| **Method; Under study design:** Survey data were collected from nursing students at a university in southern Sweden between 16–17th of January 2023.” → Consider removing exact dates unless necessary for context. | Thank you for pointing this out. We have removed the exact dates, please see the method section (page 7, line 138). |
| **Under method, instrument:** For learning methods, include a rationale for the 4-point Likert scale used (e.g., prior validation or simplification reason?). | Thank you for this insightful suggestion. We have added the sentence: “A 4-point Likert scale was used to maintain consistency with the SELC 20+20 instrument to minimize the cognitive burden for the respondents.” In the revised manuscript, please see the method section (page 8, lines 175-177). |
| **Under results**: Tables are informative but could be overwhelming due to volume.  **Suggestions:**  Consider moving **Tables 3–5** to supplementary material and summarizing key findings in the main text.  Clarify p-values with stars or bolding for significance. | Thank you for this valuable comment. We have moved Table 2-3 to supplementary material (now called S1 Table and S2 Table) and kept Table 4-5 (now called Table 2-3) in the main manuscript because these contain the main results of the study. We have clarified significant p-values with bolding. Please see the result section (pages 11-14) and the S1 Table and S2 Table. |
| **Under discussion:** Amended need:  1. The paragraph on lack of semester differences could benefit from deeper exploration (e.g., institutional teaching variation?).  2. Potential **response bias** due to voluntary participation during classroom sessions.  3. Provide clearer implications for **curriculum development** — what should be added/changed in teaching strategy?  4. Suggestion for **future longitudinal or intervention studies** to establish causality. | Thank you for raising these important aspects.  1. We have deepened the discussion about the lack of semester differences. Please see the discussion section (pages 15-16, lines 301-308). We hope this reasoning meets your expectations.  2. We acknowledge the potential risk of response bias associated with the voluntary nature of participation during classroom sessions. This limitation has now been clarified in the manuscript. Please see the discussion section (page 19, lines 388-399).  3. We have added a sentence about educational implications. Please see the discussion section (page 18, lines 363-364).  We have also added a sentence about the importance of practical training, please see the conclusion section (page 20, lines 419-420).  4. We have clarified the recommendations for future studies, please see the conclusion section (page 20, lines 421-26). |
| **Under conclusions:** Re-emphasize **educational implications** and **potential policy impact** more clearly in a final sentence. | Thank you for the helpful comment. We have tried to clarify the final sentence, please see the conclusion section (page 21, lines 426-429). |
| **Reviewer #2:** The manuscript is well written, novel, and relevant for nursing education. The response rate (89%) is commendable. Some minor points: | Thank you for your kind and encouraging comment. We truly appreciate your positive feedback on the manuscript’s quality, relevance, and response rate. It is gratifying to know that the work is considered both novel and valuable for nursing education. |
| 1. There are some ambiguity in terms of "knowledge" and "ability". The manuscript can benefit from clarifying the conceptual difference between "knowledge" and "ability" self-efficacy. E.g., knowledge = “I know what to advise,” ability = “I can conduct a lifestyle counselling session confidently”. Something along that line. | Thank you for this valuable comment. We have clarified the sentence where we explain the terms. Please see the introduction section (page 5, line 102). |
| 2. Since this study is done in one institution, what the authors would expect for other nursing students, since we have differences in curricula across institutions and countries? | Thank you for the comment. We have revised the sentence in the discussion. Please see the discussion section (page 20, lines 406-408). |
| 3. In the discussion, perhaps worth highlighting "educational implications" more concretely. Readers would be interested to learn what specific training interventions or curriculum changes would the authors recommend based on these findings? | Thank you for the important comment. We have added a sentence about educational implications. Please see the discussion section (page 18, lines 363-364). |
